# Risk Factors for 30-Day Hospital Readmission in Patients With Cancer: A Systematic Review With Emphasis on Older Adults

**DOI:** 10.7759/cureus.109309

**Published:** 2026-05-20

**Authors:** Godswill O Nwabor, Noor Subhani, Desire Weke, Reigneth C Obianenue, Happiness Olaniyi, Oyebisi M Azeez, Aliyu O Olaniyi

**Affiliations:** 1 Internal Medicine, Chesterfield Royal Hospital, Chesterfield, GBR; 2 Acute Medicine Unit, Stockport NHS Foundation Trust, Stockport, GBR; 3 General Practice, Chesterfield Royal Hospital, Chesterfield, GBR; 4 General Practice, Mersey and West Lancanshire Foundation Trust, Chesterfield, GBR; 5 Nursing, Barton Brook Care Home, Manchester, GBR; 6 Veterinary Physiology and Biochemistry, University of Ilorin, Ilorin, NGA; 7 Geriatrics, Stepping Hill Hospital, Manchester, Manchester, GBR

**Keywords:** 30-day readmission, cancer, clinical factor, comorbidity, functional status, geriatric oncology, hospital readmission, older adults, risk factors, social determinants of health

## Abstract

Thirty-day hospital readmission is a key indicator of healthcare quality, particularly in patients with cancer who are at an increased risk due to multimorbidity, treatment-related complications, and functional decline. Although older adults represent a vulnerable subgroup, evidence specific to geriatric oncology remains limited. This systematic review aimed to identify and synthesise risk factors for 30-day hospital readmission among patients with cancer, with particular emphasis on older adults.

A systematic search of MEDLINE, Embase, and the Cochrane Library (January 2015 to March 2026) was conducted in accordance with PRISMA 2020 guidelines. Eight observational studies were included. Risk factors were categorised into clinical, functional, treatment-related, and social domains. Clinical factors, including comorbidity burden and abnormal laboratory parameters (particularly hypoalbuminaemia), were the most consistently reported predictors. Functional impairment and geriatric-specific factors were strongly associated with the risk of readmission in studies focusing on older adults. Treatment-related factors such as prolonged length of stay and polypharmacy, along with social determinants including limited social support, further contributed to increased risk.

Considerable heterogeneity across study populations, methodologies, and outcome reporting precluded meta-analysis. Overall, the evidence suggests that readmission risk in patients with cancer is multifactorial, with consistent direction of association across studies despite variability in magnitude. However, the limited number of geriatric-specific studies highlights a critical gap in the literature.

These findings support the need for multidimensional risk assessment and targeted transitional care interventions. Future research should prioritise prospective, geriatric-focused studies and the development of validated risk prediction models.

## Introduction and background

Hospital readmission within 30 days of discharge is widely recognised as an important indicator of healthcare quality, continuity of care, and system efficiency [1]. In oncology populations, readmissions are common because cancer treatment frequently involves complex multimodal interventions, including chemotherapy, radiotherapy, surgery, and immunotherapy, which may result in treatment-related complications and clinical instability requiring rehospitalisation. Approximately 20-30% of oncology patients are readmitted within 30 days of discharge, contributing substantially to healthcare burden, increased costs, and interruptions in treatment continuity [2,3].

Older adults represent a particularly vulnerable subgroup, accounting for more than 60% of cancer diagnoses [4]. This population commonly experiences multimorbidity (the presence of multiple chronic conditions), polypharmacy (the use of multiple medications), frailty, cognitive impairment, and reduced physiological reserve, all of which increase susceptibility to adverse outcomes, including hospital readmission [5]. Frailty refers to a state of increased vulnerability resulting from age-related decline across multiple physiological systems, leading to reduced resilience to stressors and poorer clinical outcomes. Functional decline and geriatric syndromes may further complicate recovery following discharge, highlighting the importance of comprehensive geriatric assessment (CGA), a multidimensional evaluation of medical, functional, cognitive, psychological, and social needs in older adults, in identifying vulnerabilities and guiding individualised care strategies in older patients with cancer [6,7].

Despite growing recognition of the importance of preventing readmission, important gaps remain in the existing literature. Most studies examine broad oncology populations rather than geriatric-specific cohorts, limiting understanding of how functional impairment, geriatric syndromes, and social vulnerability influence readmission risk in older adults with cancer. In addition, the available evidence is heterogeneous across cancer types, treatment settings, and study methodologies, reducing generalisability across oncology populations. Prior research has also focused predominantly on clinical predictors, while functional status and social determinants of health remain comparatively underexplored despite emerging evidence suggesting their contribution to readmission risk [5,8]. Because postoperative readmissions are influenced by distinct perioperative and surgical recovery factors, this review focused specifically on non-surgical oncology readmissions to improve comparability across studies and better evaluate medical, functional, and social predictors of readmission risk.

Although several predictive models for oncology readmission have been proposed, many remain limited by insufficient external validation, restricted generalisability across healthcare systems, and inconsistent incorporation of geriatric-specific variables and social determinants of health [2,9]. Consequently, their clinical applicability in geriatric oncology populations remains uncertain. To address these gaps, this systematic review focuses on risk factors associated with 30-day hospital readmission among patients with cancer, with particular emphasis on older adults. Using a multidimensional framework, this review synthesises evidence relating to clinical, functional, treatment-related, and social determinants of readmission risk and identifies priorities for future geriatric oncology research [9].

## Review

Study design and reporting

This systematic review was conducted in accordance with the PRISMA 2020 guidelines to ensure methodological rigour, transparency, and reproducibility [10]. A predefined internal protocol guided all stages of the review process, including study identification, screening, eligibility assessment, data extraction, and synthesis. The review was not prospectively registered in PROSPERO because substantial stages of the review process had already commenced before registration was sought, rendering the review ineligible for prospective registration according to PROSPERO guidance. Nevertheless, predefined methods were consistently followed throughout the review to minimise reporting bias and maintain methodological consistency. The protocol is available from the corresponding author upon reasonable request.

Conceptual framework and search strategy

The search strategy was developed using a Population-Exposure-Comparator-Outcome (PECO) framework to ensure a structured and comprehensive identification of relevant studies. The Population (P) included adult patients with cancer, with particular emphasis on older adults (≥65 years). The Exposure (E) comprised risk factors or predictors associated with hospital readmission, while the Comparator (C) was implicitly defined as patients without or with lower exposure to these risk factors, consistent with observational study frameworks. The Outcome (O) was 30-day hospital readmission [11].

Based on this framework, four key conceptual domains were identified: cancer, older adults, hospital readmission, and risk factors. These domains guided the development of the search strategy to capture studies evaluating predictors of early readmission among geriatric oncology populations. To maximise sensitivity and ensure comprehensive retrieval, both controlled vocabulary (e.g., Medical Subject Headings [MeSH]) and free-text terms were used. Search terms within each domain were combined using the Boolean operator “OR,” while the four domains were combined using “AND” to enhance specificity and relevance. Detailed database-specific search strategies are provided in the Appendices.

The core search strategy was structured as follows: (cancer OR neoplasm OR malignancy OR tumour OR oncology) AND (elderly OR older adults OR geriatric OR aged) AND (hospital readmission OR 30-day readmission OR rehospitalisation) AND (risk factors OR predictors OR determinants OR associated factors).

This structured PECO-based approach facilitated a comprehensive and systematic retrieval of relevant literature across multiple databases (Table 1).

**Table 1:** PECO framework used in the study Adapted from Morgan et al. (2018) [11] PECO, Population, Exposure, Comparator, Outcome

Component	Description
Population (P)	Adults with cancer, with particular focus on older adults (≥65 years), including both geriatric-specific and broader oncology populations
Exposure (E)	Risk factors associated with 30-day hospital readmission
Comparator (C)	Patients without the identified risk factors or with lower levels of exposure to these predictors
Outcome (O)	30-day hospital readmission
Study design	Observational analytical studies (retrospective cohort and case-control studies)

Information sources

A comprehensive literature search was conducted using electronic databases accessed through the hospital library, including MEDLINE (Ovid), Embase, and the Cochrane Library. Searches covered database from inception to March 2026, with inclusion restricted to studies published in English between January 2015 and March 2026. The final search was conducted between 1 March 2026 and 31 March 2026. Search strategies combined controlled vocabulary terms (e.g., MeSH and Emtree terms) with free-text keywords related to cancer, older adults, hospital readmission, and risk factors. Boolean operators (“AND” and “OR”) were used to optimise sensitivity and specificity. Filters were applied, where appropriate, to restrict studies to human adult populations and observational analytical study designs. To ensure completeness, additional studies were identified through manual screening of the reference lists of included articles and relevant review papers [10-12]. Full database-specific search strategies and search yields are provided in Appendices A-C.

Study selection

The study selection process was conducted systematically in accordance with predefined inclusion and exclusion criteria. A total of 3,281 records were identified, including 3,270 records from database searching (Embase: 2,539; MEDLINE: 711; Cochrane Library: 20) and 11 additional records identified through reference screening. After removal of 542 duplicate records, 2,739 unique studies remained and were imported into the Rayyan software (Rayyan Systems Inc., Cambridge, MA) for title and abstract screening.

Screening was performed independently by two reviewers to ensure consistency and minimise selection bias. During the initial screening phase, 2,570 records were excluded based on irrelevance, leaving 169 articles for full-text assessment. All full-text articles were retrieved and evaluated for eligibility. Of these, 99 studies were excluded for not meeting the inclusion criteria. The remaining 70 studies underwent further detailed assessment, resulting in the exclusion of an additional 62 studies.

**Figure 1:**
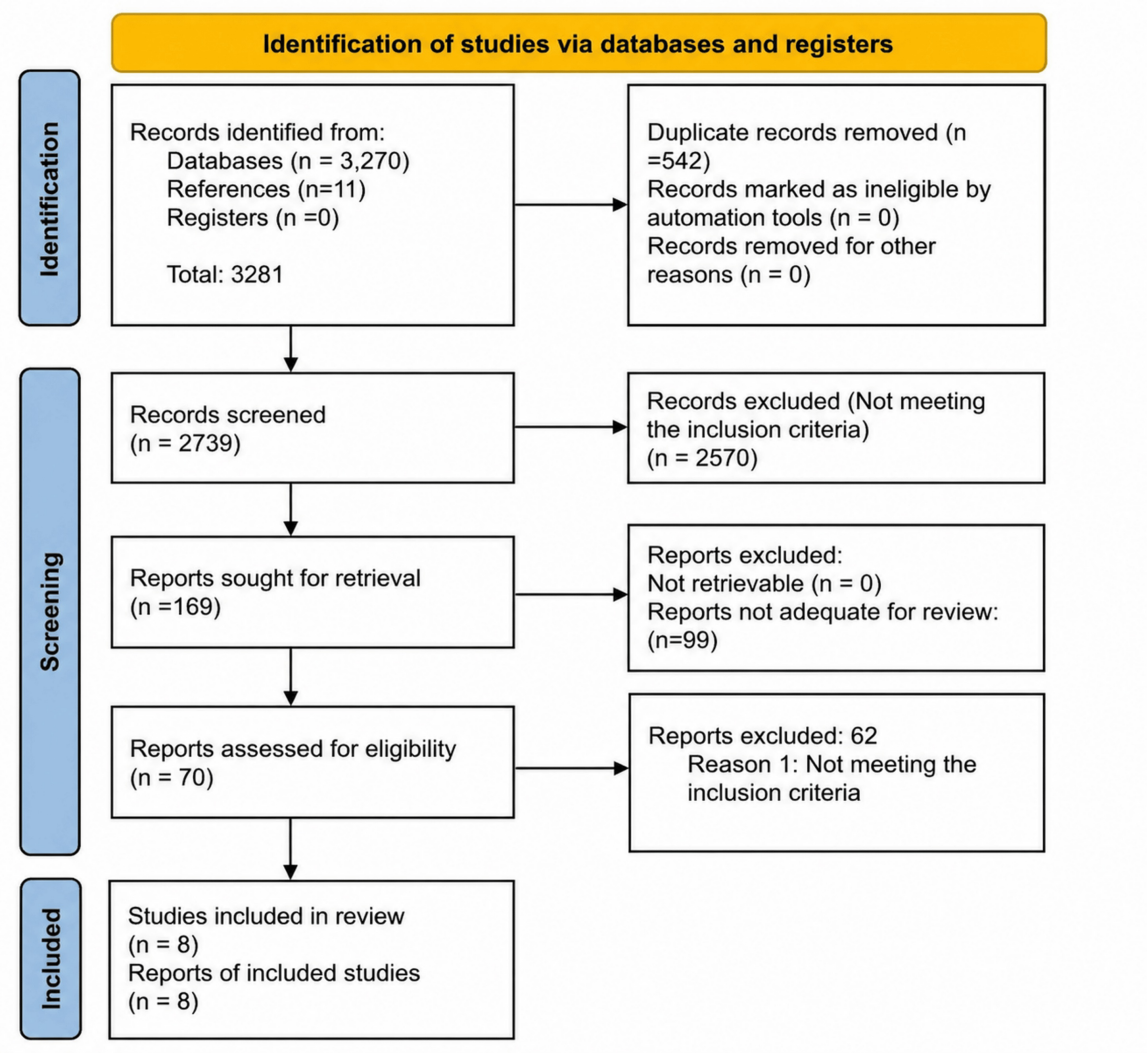
PRISMA 2020 flow diagram for the study selection process PRISMA, Preferred Reporting Items for Systematic Reviews and Meta-Analyses

Ultimately, eight studies [2,3,9,13-17] met all eligibility criteria and were included in the final qualitative synthesis. Any disagreements between reviewers during the selection process were resolved through discussion and consensus, with a third senior reviewer consulted when necessary. The overall study selection process is illustrated in the PRISMA flow diagram (Figure 1).

Data extraction

Data extraction was conducted independently by two reviewers using a standardised data extraction form developed for this review. Extracted variables included study characteristics (author, year, country, study design, and sample size), patient population, cancer type, definitions of 30-day readmission, identified risk factors, statistical methods, and key findings including reported effect estimates where available. Disagreements during data extraction were resolved through discussion and consensus, with consultation from a third reviewer when necessary.

Eligibility criteria

Studies were eligible for inclusion if they involved adult patients with cancer, with particular relevance to older adults, and examined risk factors associated with 30-day hospital readmission. Only observational analytical study designs, including cohort and case-control studies, were included. Eligible studies were required to be published in English in peer-reviewed journals between 2015 and 2026. Studies were excluded if they involved non-cancer populations, did not report 30-day readmission outcomes, focused primarily on surgical or postoperative readmissions, were non-analytical in design such as reviews or editorials, or examined highly specialised populations such as intensive care cohorts or specific experimental treatment settings. Surgical and postoperative readmission studies were excluded because postoperative complications represent a distinct clinical pathway characterised by procedure-specific risk factors, perioperative care processes, and surgical recovery trajectories that differ substantially from medical oncology admissions. Excluding these studies reduced clinical heterogeneity and improved comparability across included studies.

Study characteristics

A total of eight studies were included in the review (Table 2). The included studies comprised observational designs, including retrospective cohort and case-control studies, conducted across multiple countries, predominantly the United States, with one study conducted in China [2,3,9,13-17). Sample sizes varied substantially, ranging from 257 to 32,457 participants. Two studies specifically focused on older adults aged 65 years and above [9,13], while the remaining studies included broader oncology populations or specific cancer subgroups such as head and neck cancer, breast cancer, or malignancy-related complications [2,3,14-17]. All studies examined factors associated with 30-day hospital readmission using hospital or administrative data and multivariable statistical analyses.

**Table 2:** Characteristics of included studies examining risk factors for 30-day hospital readmission in patients with cancer

Study	Country	Design	Population	Sample Size	Outcome	Key Findings
Stabellini et al., 2023 [2]	USA	Retrospective cohort study using machine learning models	Adult patients with cancer across multiple tumour types	Large database	30-day readmission	Prior hospital utilisation, comorbidity burden, and treatment-related variables. Importantly, social determinants of health
Tian et al. (2024) [3]	China	Retrospective cohort	Patients with malignancy-related ascites	32,457	30-day readmission	Comorbidities and disease complications increased risk
Burhenn et al. (2020) [9]	USA	Case-control	Older adults (≥65) with solid tumours	368	30-day readmission	Abnormal lab values (hemoglobin, sodium, albumin) predicted readmission
Chiang et al. (2015) [13]	USA	Retrospective cohort	Older adults (≥65) with cancer	677	30-day readmission	Functional dependence and geriatric factors increased risk
Donzé et al. (2017) [14]	USA/Switzerland	Retrospective cohort	Adult cancer patients	2,916	30-day readmission	Polypharmacy, liver disease, low sodium and hemoglobin were predictors
Manzano et al. (2022) [16]	USA	Retrospective cohort	Solid tumour patients	6,720	30-day readmission	Length of stay, anemia, metastasis, and social factors predicted readmission
Tennison et al. (2021) [15]	USA	Retrospective cohort	Cancer patients post-rehabilitation	257	30-day readmission	Low mobility, increased medications, and low hemoglobin increased risk
Mqadi et al., 2026 [17]	South Africa	Retrospective study using machine learning models	Adult patients with breast cancer	257	30-day hospital readmission	Comorbidities, treatment-related variables, and prior healthcare utilisation

Risk-of-Bias Assessment

The methodological quality of the included studies was assessed using the Newcastle-Ottawa Scale (NOS) for observational studies (Table 3) [18]. This tool evaluates studies across three domains: selection of participants (maximum 4 stars), comparability of study groups (maximum 2 stars), and outcome assessment (maximum 3 stars).

**Table 3:** Risk-of-bias assessment of included studies using the Newcastle-Ottawa Scale

Study	Selection (Max 4)	Comparability (Max 2)	Outcome (Max 3)	Total Score (Max 9)	Risk of Bias
Stabellini et al., 2023 [2]	4	1	3	8/9	Low
Tian et al., 2024 [3]	4	1	3	8/9	Low
Burhenn et al., 2020 [9]	4	1	3	8/9	Low
Chiang et al., 2015 [13]	4	1	3	8/9	Low
Donzé et al., 2017 [14]	4	1	3	8/9	Low
Manzano et al., 2022 [16]	3	1	3	7/9	Low
Tennison et al., 2021 [15]	3	1	3	7/9	Low
Mqadi et al., 2026 [17]	3	1	3	7/9	Low

Studies were classified based on NOS scores as follows: 7-9 stars indicated low overall risk of bias, 4-6 stars indicated moderate risk, and fewer than 4 stars indicated high risk of bias, in accordance with established methodological guidance [18]. Overall NOS scores ranged from 7 to 8 out of a maximum of 9, indicating generally low methodological risk across the included studies.

Most studies demonstrated a low risk of bias in outcome assessment, as 30-day readmission was clearly defined and consistently measured using hospital records or administrative datasets [2,3,9,13,14]. The use of objective outcome measures strengthens the reliability of findings across studies. Although all included studies achieved NOS scores consistent with low overall risk of bias, several retrospective studies remained vulnerable to residual confounding, selection bias, and incomplete adjustment for functional and social determinants [15,16,17].

Comparability across studies was variable. While some studies adjusted for key clinical confounders such as comorbidity burden and prior healthcare utilisation, fewer accounted for functional status or social determinants of health, which are increasingly recognised as important predictors of readmission. In addition, variation in sample size, study design, cancer populations, and healthcare settings may have influenced methodological quality and limited external validity.

Despite these limitations, the overall methodological quality of the included studies was considered adequate to support the synthesis of key risk factors. Nevertheless, the findings should be interpreted with caution, given the potential for residual confounding, retrospective study design limitations, and heterogeneity across studies.

Data synthesis

Due to substantial clinical and methodological heterogeneity across the included studies, including variations in study design, patient populations, cancer types, and outcome definition, a meta-analysis was not considered appropriate. Instead, the findings were synthesised narratively, with emphasis placed on the consistency, direction, and strength of associations reported across studies.

Risk factors for 30-day hospital readmission

Across the included studies, several consistent predictors of 30-day hospital readmission were identified, highlighting the multifactorial and complex nature of readmission risk among patients with cancer. Risk factors were grouped into four domains: clinical, functional, treatment-related, and social. Despite variability in study populations, settings, and methodologies, the direction of associations was largely consistent across studies, although the magnitude of effects varied.

Clinical factors demonstrated the strongest and most consistent level of evidence. A higher comorbidity burden and abnormal laboratory parameters, particularly hypoalbuminaemia, anaemia, and hyponatraemia, were strongly associated with increased readmission risk across multiple studies [2,9,14-16]. These associations were consistently reported in both large cohort studies and smaller analyses, indicating high confidence in the robustness of these findings. Although effect sizes varied, the direction of association remained uniform, supporting clinical instability as a primary driver of readmission.

Clinical instability demonstrated not only consistent direction of association but also clinically meaningful effect sizes across studies. Burhenn et al. reported that older adults with cancer who had at least two abnormal laboratory parameters, including haemoglobin, albumin, sodium, and SGOT (serum glutamic-oxaloacetic transaminase), were approximately three times more likely to experience 30-day readmission compared with patients with one or no abnormal laboratory values [9]. Similarly, Manzano et al. identified prolonged length of stay, anaemia, metastatic disease, and prior healthcare utilisation as significant contributors within the validated Cancer READMIT prediction model [16]. Donzé et al. further demonstrated that neoplastic disease was strongly associated with potentially avoidable readmission (OR 5.60, 95% CI 2.85-10.98), while opiate use at discharge increased readmission odds more than two-fold (OR 2.29, 95% CI 1.29-4.07) [14]. Although effect estimates varied because of differences in study design, patient populations, and adjustment strategies, the consistency of associations across studies strengthens confidence in these predictors.

Functional factors showed a moderate but consistent level of evidence, particularly in older populations. Impairments in activities of daily living, reduced mobility, and decreased functional capacity were associated with increased readmission risk [9,14,15]. However, the limited number of studies explicitly incorporating geriatric assessments reduces the overall strength of evidence. While the direction of association was consistent, the smaller evidence base limits confidence in generalisability.

Treatment-related factors demonstrated moderate evidence with consistent direction of association. Prolonged length of hospital stay, polypharmacy, and clinical instability at discharge were associated with increased risk of readmission [14-16]. These findings were reported across multiple studies, although variability in healthcare systems and discharge practices may influence the magnitude of these associations.

Social determinants of health showed emerging but lower-strength evidence. Factors such as socioeconomic status, race, and access to social support were associated with readmission risk when reported [2,16,17]. However, these variables were inconsistently measured and reported across studies, limiting the overall strength of evidence. Studies employing machine learning approaches were more likely to identify these complex interactions, although these models often lacked external validation.

Overall, the consistency in the direction of associations across studies supports a multifactorial model of readmission risk, with clinical factors demonstrating the highest level of evidence, followed by functional and treatment-related factors, and social determinants representing an emerging but underexplored domain.

The certainty of evidence across domains was assessed using a GRADE (Grading of Recommendations Assessment, Development and Evaluation) adapted GRADE-informed certainty framework (Table 4), taking into account study design, consistency of findings, and methodological limitations. Clinical factors demonstrated moderate certainty of evidence, functional and treatment-related factors were supported by low-certainty evidence, and social determinants were supported by very low-certainty evidence. This graded synthesis highlights the relative strength and consistency of risk factors across clinical, functional, treatment-related, and social domains, providing a structured interpretation of the available evidence. Because this review focused on prognostic and risk-factor evidence rather than interventions, the framework was applied descriptively to assess consistency, methodological limitations, and overall confidence in the evidence across domains.

**Table 4:** GRADE summary of evidence for risk factors associated with 30-day hospital readmission in patients with cancer ADL, activities of daily living; GRADE, Grading of Recommendations Assessment, Development, and Evaluation

Domain	Key Risk Factors	Consistency of Evidence	Certainty of Evidence (GRADE)	Reasons for Downgrading
Clinical factors	Comorbidity burden, hypoalbuminaemia, anaemia, hyponatraemia	High consistency across studies [2,9,14,15,16]	Moderate	Observational design, risk of confounding
Functional factors	Impaired ADLs, reduced mobility, functional decline	Moderate consistency [9,14,15]	Low	Limited number of studies, inconsistent measurement
Treatment-related factors	Length of stay, polypharmacy, and discharge instability	Moderate consistency [14,15,16]	Low	Heterogeneity in healthcare systems, residual confounding
Social determinants	Socioeconomic status, social support, access to care	Low–moderate consistency [2,16,17]	Very Low	Inconsistent reporting, lack of standardisation, limited data

Table 5 presents an adapted GRADE-informed assessment of the certainty of evidence across major risk-factor domains identified in the review. Because the included studies were observational, prognostic, and risk-factor studies rather than intervention studies, the framework was applied descriptively to evaluate methodological limitations, consistency of findings, indirectness, imprecision, and potential publication bias across domains. Overall certainty ratings were categorised as moderate, low, or very low based on the collective strength and consistency of the available evidence [18].

**Table 5:** Adapted GRADE-informed certainty assessment across risk factor domains for 30-day hospital readmission in patients with cancer GRADE, Grading of Recommendations Assessment, Development, and Evaluation

Domain	Risk of Bias	Inconsistency	Indirectness	Imprecision	Publication Bias	Overall Certainty
Clinical factors	Low concern	Low	Low	Moderate	Possible	Moderate
Functional factors	Moderate	Moderate	Moderate	Moderate	Possible	Low
Treatment-related factors	Moderate	Moderate	Moderate	Moderate	Possible	Low
Social determinants	Moderate	High	High	High	Likely	Very low

Discussion

This systematic review provides a structured and graded synthesis of risk factors associated with 30-day hospital readmission in patients with cancer, with particular emphasis on older adults. The findings confirm that readmission risk is multifactorial, involving a complex interplay of clinical, functional, treatment-related, and social determinants. Importantly, this review extends beyond descriptive reporting by evaluating the relative strength and consistency of evidence across these domains.

Clinical factors demonstrated the strongest and most consistent evidence across studies. Comorbidity burden and abnormal laboratory parameters, particularly hypoalbuminaemia, anaemia, and hyponatraemia, were repeatedly associated with increased readmission risk [2,9,14,16]. The consistency of these findings across diverse study designs and populations supports confidence in their role as important predictors of readmission. These factors likely reflect unresolved physiological instability at discharge. Hypoalbuminaemia, in particular, represents a biologically plausible marker of both malnutrition and systemic inflammation, both of which are associated with poorer outcomes in cancer populations. Although the direction of association was consistent, variation in effect size may reflect differences in disease severity, cancer subtype, and patient characteristics across studies [2,9,19]. The observed magnitude of association in several included studies further supports the clinical relevance of these predictors. For example, Burhenn et al. demonstrated an approximately threefold increase in readmission risk among patients with multiple abnormal laboratory parameters, while Donzé et al. reported substantially increased odds of avoidable readmission among patients with neoplastic disease and opiate exposure at discharge [9,14]. These findings suggest that markers of physiological instability and treatment complexity may have important predictive relevance in oncology populations.

Functional factors demonstrated moderate but clinically important evidence, particularly in studies focusing on older adults. Impairments in activities of daily living and reduced mobility were consistently associated with increased readmission risk [9,13,15]. These findings highlight the importance of geriatric-specific factors, as reduced functional reserve may limit patients’ ability to manage post-discharge complications and maintain treatment adherence. However, the limited incorporation of CGA across studies represents a significant gap in the literature. Integrating CGA into oncology practice may improve risk stratification by capturing domains such as frailty, cognitive impairment, nutritional vulnerability, and functional decline [6,7,9].

Treatment-related factors also demonstrated moderate evidence with consistent direction of association. Prolonged length of stay, polypharmacy, and discharge-related instability were associated with increased likelihood of readmission [14-16]. These variables likely reflect both disease severity and healthcare system processes, including the quality of discharge planning and transitional care. Differences in healthcare systems, discharge practices, and supportive care resources across studies may partly explain variation in the magnitude of these associations. Transitions of care represent a particularly vulnerable period, and inadequate discharge planning has been linked to increased readmission risk, especially among patients with complex medical and functional needs [1,20].

Social determinants of health, although less consistently reported, demonstrated a clear direction of association when included. Factors such as socioeconomic disadvantage, limited social support, and reduced access to follow-up care were associated with increased readmission risk [2,16,17]. However, the overall strength of evidence remains limited because these variables were inconsistently measured and underreported across studies. Studies employing machine learning approaches were more likely to identify complex interactions between social and clinical variables; however, many of these models lack robust external validation, limiting their immediate clinical applicability in geriatric oncology populations [2,17,21]. Existing prediction models also inadequately incorporate frailty, functional decline, and social vulnerability, despite the recognised importance of these factors in older adults with cancer. These findings highlight an important gap in the literature and underscore the need for more comprehensive integration of social and geriatric factors into future predictive models and readmission research.

The graded synthesis of evidence in this review has important clinical implications. Clinical factors appear to represent the most consistently supported predictors and should be prioritised in risk stratification efforts, while functional and treatment-related factors provide additional important context, particularly in older adults. Social determinants, although currently supported by lower-certainty evidence, represent a critical and underexplored domain that may substantially influence post-discharge outcomes and healthcare disparities.

Despite these insights, several limitations should be considered. The evidence base remains limited, with only a small number of studies specifically focusing on geriatric oncology populations. Inclusion of broader oncology cohorts was necessary because of the scarcity of geriatric-specific data but may limit the direct applicability of findings to older adults with cancer. Furthermore, heterogeneity in study design, patient populations, healthcare settings, and outcome definitions precluded formal meta-analysis. Nevertheless, the consistency in the direction of associations across studies supports the overall validity of the findings despite these methodological limitations.

In summary, this review demonstrates that 30-day hospital readmission in patients with cancer is driven by a complex and interrelated set of clinical, functional, treatment-related, and social factors. Clinical predictors demonstrated the strongest and most consistent associations, followed by functional and treatment-related factors, while social determinants remain an emerging but underexplored area of evidence. Addressing these risks will require a comprehensive, multidimensional approach to oncology care, particularly in geriatric populations. Future research should prioritise prospective, high-quality studies integrating clinical, functional, and social domains, alongside the development and external validation of robust and generalisable readmission prediction models tailored to older adults with cancer [1,2,9,21].

Clinical implications

The findings of this review have several important implications for clinical practice. First, clinical factors such as comorbidity burden and abnormal laboratory parameters represent high-confidence predictors and should be prioritised in risk stratification at the point of discharge. Early identification of physiologically unstable patients may allow for targeted interventions to reduce readmission risk.

Secondly, the importance of functional status, particularly in older adults, highlights the need for routine integration of CGA into oncology care. Assessing domains such as mobility, functional independence, and frailty may improve identification of high-risk patients and guide personalised care planning [6,7].

Thirdly, treatment-related factors, including polypharmacy and prolonged hospitalisation, emphasise the need for structured discharge planning and transitional care strategies. Interventions such as medication reconciliation, early follow-up, and multidisciplinary discharge planning may help mitigate preventable readmissions [1, 20].

Finally, although supported by lower-strength evidence, social determinants of health play a critical role in post-discharge outcomes. Addressing factors such as social support and access to care may improve continuity of care and reduce disparities in readmission risk. Future clinical pathways should incorporate these multidimensional factors to optimise patient outcomes [20].

Limitations

This review has several limitations. First, the small number of included studies (n = 8), together with their predominantly retrospective design, limits the overall strength of the evidence and precludes causal inference [22]. Second, substantial heterogeneity in study populations, cancer types, study methodologies, and outcome definitions reduced comparability across studies and prevented formal meta-analysis. Third, although the review focused on older adults, many included studies examined broader oncology populations rather than exclusively geriatric cohorts, thereby limiting the direct applicability of findings to older patients with cancer. Restriction to English-language publications and exclusion of grey literature may also have introduced language and publication bias, potentially limiting inclusion of relevant unpublished or region-specific evidence and reducing the global generalisability of findings. In addition, inter-rater reliability statistics such as Cohen’s kappa were not formally calculated during screening, which may limit assessment of reviewer agreement despite the use of independent duplicate screening procedures. The absence of prospective protocol registration (e.g., PROSPERO) may also increase the risk of reporting bias, although a predefined protocol was followed throughout the review process to maintain methodological consistency. Accordingly, these limitations warrant cautious interpretation of the findings and underscore the need for further high-quality, prospective research in geriatric oncology populations.

## Conclusions

This systematic review identified several multidimensional factors associated with 30-day hospital readmission among patients with cancer, particularly older adults. Clinical predictors, including comorbidity burden, hypoalbuminaemia, anaemia, and hyponatraemia, demonstrated the strongest and most consistent associations with readmission risk. Functional impairment, reduced mobility, and geriatric vulnerability were also important predictors, particularly in older populations.

Treatment-related factors such as prolonged hospitalisation, polypharmacy, and discharge instability contributed substantially to the risk of readmission. At the same time, social determinants, including limited social support and reduced access to care, emerged as important but underexplored contributors. Although the included studies generally demonstrated low risk of bias, methodological heterogeneity limited direct comparability and precluded meta-analysis.

These findings support the need for multidimensional risk assessment, comprehensive geriatric evaluation, and improved transitional care strategies to reduce avoidable readmissions in oncology populations. Future research should prioritise prospective geriatric-focused studies and externally validated prediction models integrating clinical, functional, and social determinants of health.

## Appendices

Appendix A 

**Table 6:** Embase Search Strategy (January 1974 to March 2026)

Step	Search Query	Results
1	exp Neoplasms/	#######
2	Cancer OR Neoplasm* OR Malignanc* OR Oncolog* OR Tumor* OR Tumour*	#######
3	1 OR 2	#######
4	exp Aged/	#######
5	Older Adult* OR Elderly OR Ageing OR Geriatric* OR Older people OR Aging	#######
6	4 OR 5	#######
7	exp Patient Readmission/	1,34,620
8	Readmission OR Re-hospitalisation OR Re-hospitalization	1,47,525
9	30 day* OR Thirty-day*	3,25,702
10	8 AND 9	42,251
11	Readmission adj4 (30 day* OR Thirty-day*)	23,262
12	7 OR 11	1,35,935
13	3 AND 6 AND 12	10,083
14	exp Risk Factors/	#######
15	Predictor* OR Associated factor* OR Determinant* OR Prognostic factor* OR Risk factor*	#######
16	14 OR 15	#######
17	13 AND 16	2,539

Appendix B 

**Table 7:** MEDLINE (Ovid) Search Strategy (January 1946 to March 2026)

Step	Search Query	Results
1	exp Neoplasms/	#######
2	Cancer OR Neoplasm* OR Malignanc* OR Oncolog* OR Tumor* OR Tumour*	#######
3	1 OR 2	#######
4	exp Aged/	#######
5	Older Adult* OR Elderly OR Ageing OR Geriatric* OR Older people OR Aging	#######
6	4 OR 5	#######
7	exp Patient Readmission/	27,132
8	Readmission OR Re-hospitalisation OR Re-hospitalization	54,950
9	30 day* OR Thirty-day*	1,85,289
10	8 AND 9	17,846
11	Readmission adj4 (30 day* OR Thirty-day*)	11,199
12	7 OR 11	32,578
13	3 AND 6 AND 12	2,177
14	exp Risk Factors/	#######
15	Predictor* OR Associated factor* OR Determinant* OR Prognostic factor* OR Risk factor*	#######
16	14 OR 15	#######
17	13 AND 16	711

Appendix C 

**Table 8:** Cochrane Library Search Strategy

Step	Search Query	Results
1	MeSH: Neoplasms	1,32,882
2	Cancer OR Neoplasm* OR Malignanc* OR Oncolog* OR Tumor* OR Tumour*	2,98,549
3	#1 OR #2	3,12,635
4	MeSH: Aged	2,91,569
5	Older Adult* OR Elderly OR Ageing OR Geriatric* OR Older people OR Aging	1,07,944
6	#4 OR #5	3,63,812
7	MeSH: Patient Readmission	1,728
8	Readmission NEAR/4 (30 day* OR Thirty-day*)	2,221
9	#7 OR #8	3,723
10	#3 AND #6 AND #9	140
11	MeSH: Risk Factors	38,749
12	Predictor* OR Associated factor* OR Determinant* OR Prognostic factor* OR Risk factor*	1,47,082
13	#11 OR #12	1,47,089
14	#10 AND #13	20
